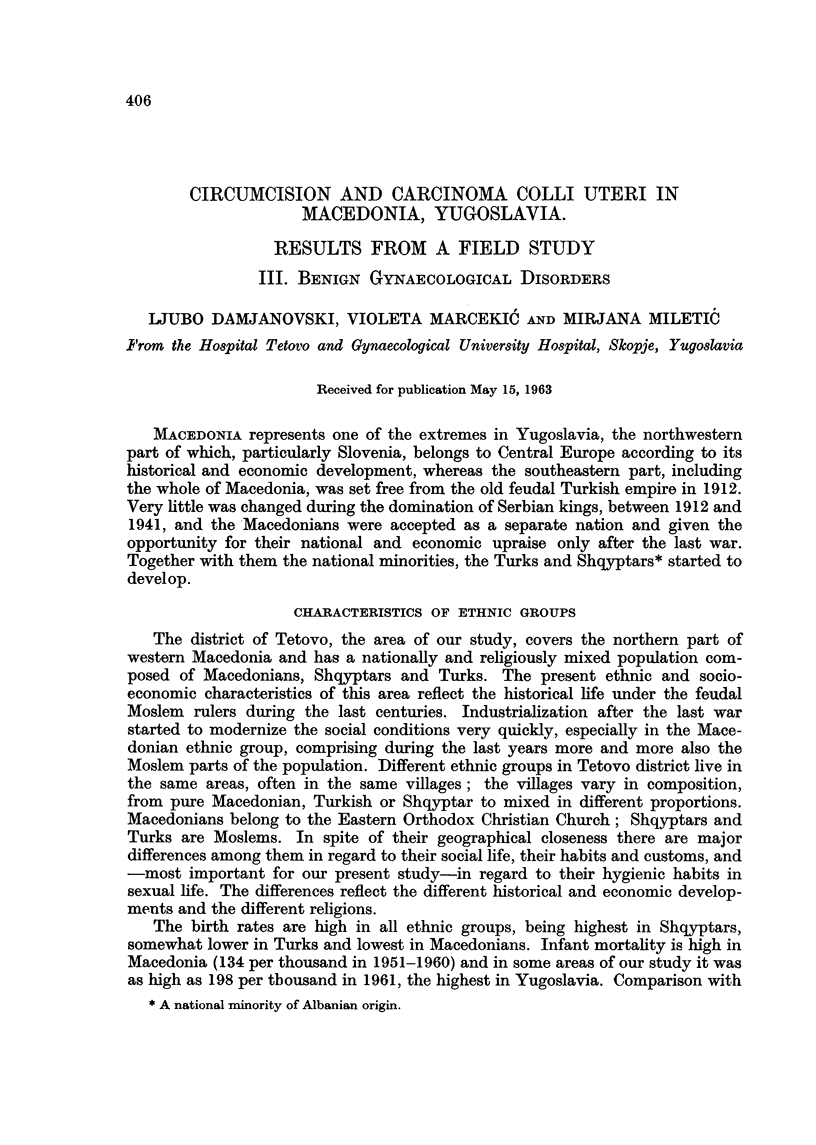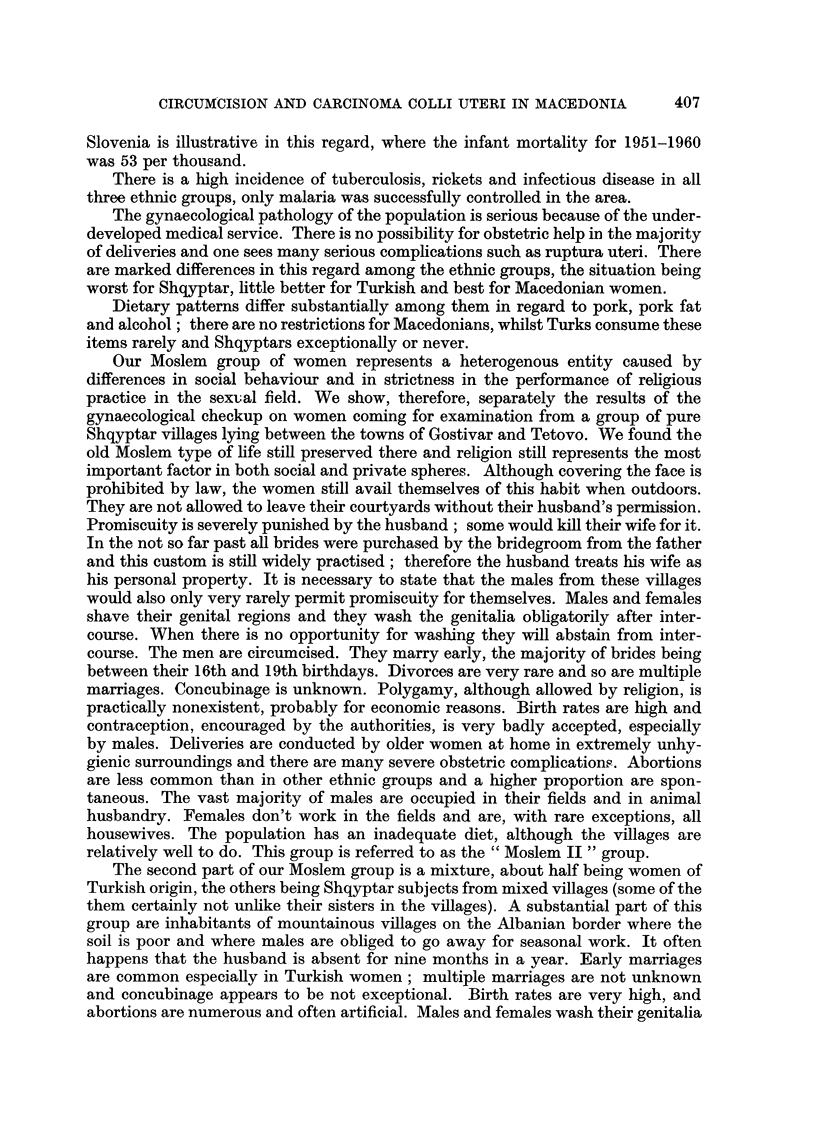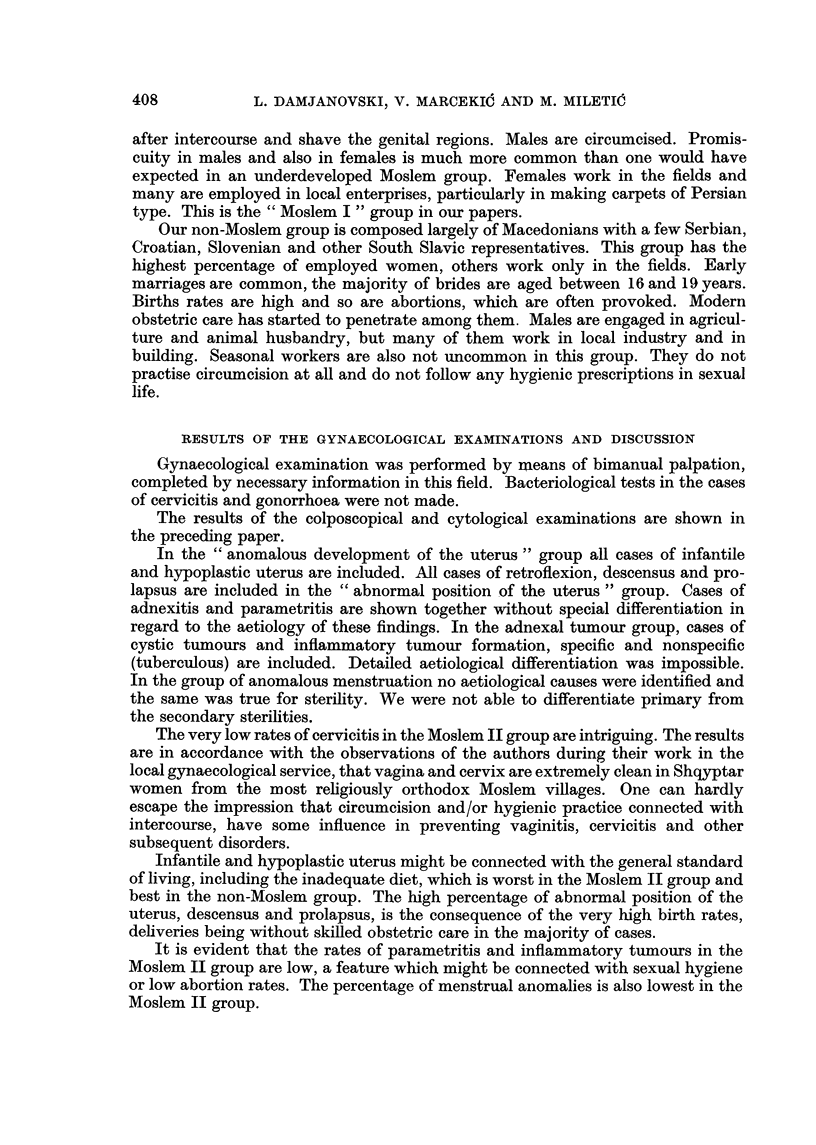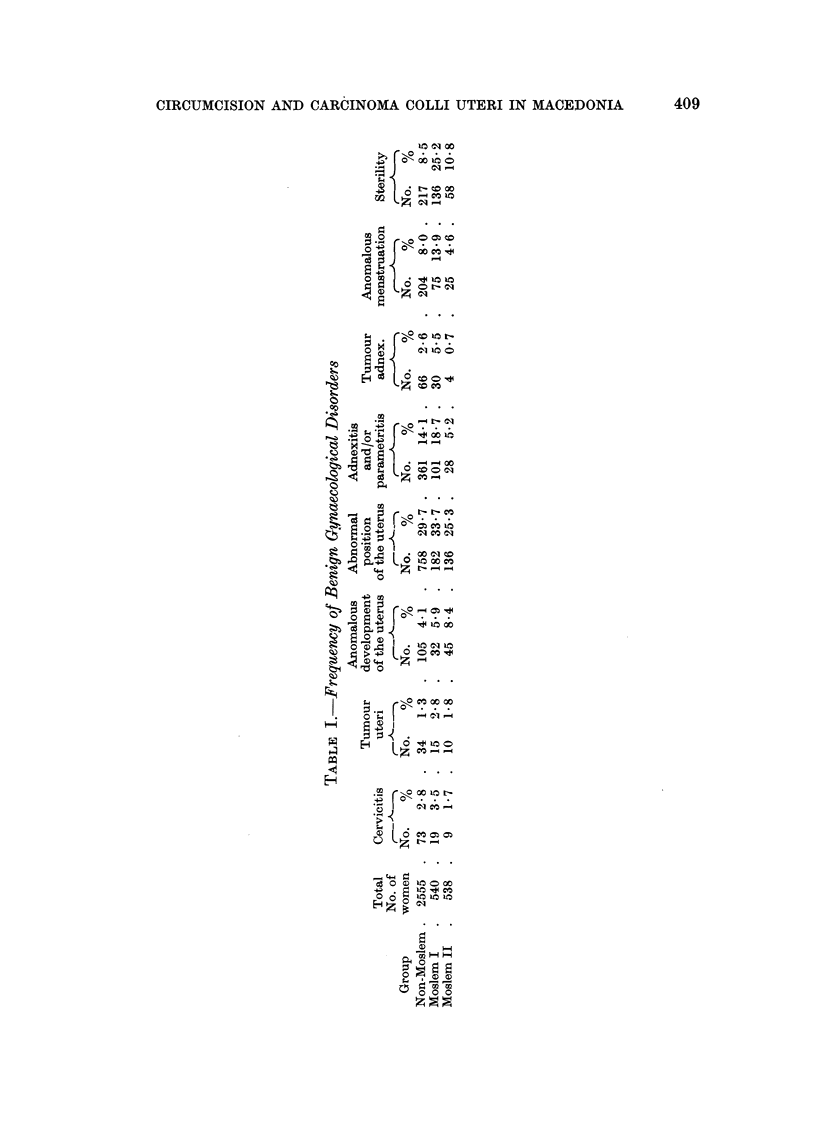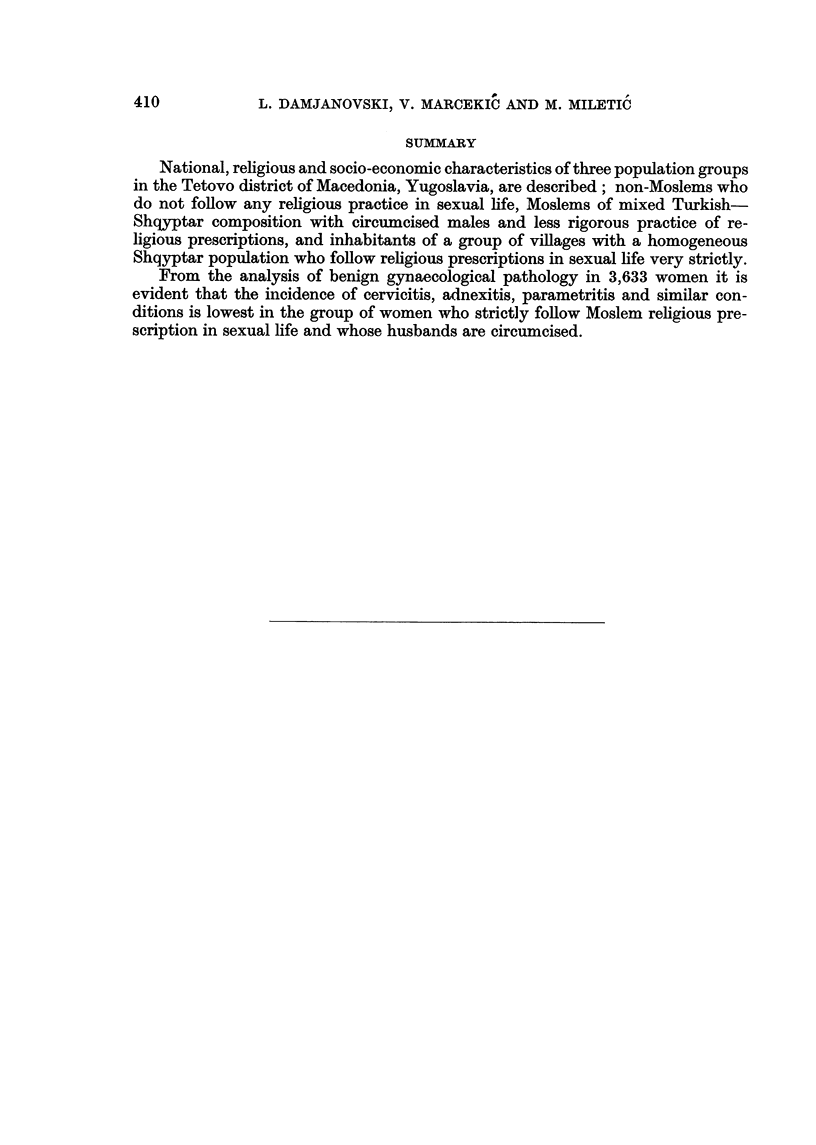# Circumcision and Carcinoma Colli Uteri in Macedonia, Yugoslavia. Results from a Field Study

**DOI:** 10.1038/bjc.1963.56

**Published:** 1963-09

**Authors:** Ljubo Damjanovski, Violeta Marcekić, Mirjana Miletić


### CIRCUMCISION AND CARCINOMA COLLI UTERI IN MACEDONIA, YUGOSLAVIA.

### RESULTS FROM A FIELD STUDY

### III. Benign Gynaecological Disorders

LJUBO DAMJANOVSKI, VIOLETA MARCEKIĆ AND MIRJANA MILETIĆ

*From the Hospital Tetovo and Gynaecological University Hospital, Skopje, Yugoslavia*

Received for publication May 15, 1963

Macedonia represents one of the extremes in Yugoslavia, the northwestern part of which, particularly Slovenia, belongs to Central Europe according to its historical and economic development, whereas the southeastern part, including the whole of Macedonia, was set free from the old feudal Turkish empire in 1912. Very little was changed during the domination of Serbian kings, between 1912 and 1941, and the Macedonians were accepted as a separate nation and given the opportunity for their national and economic upraise only after the last war. Together with them the national minorities, the Turks and Shqyptars* started to develop.

CHARACTERISTICS OF ETHNIC GROUPS

The district of Tetovo, the area of our study, covers the northern part of western Macedonia and has a nationally and religiously mixed population composed of Macedonians, Shqyptars and Turks. The present ethnic and socio-economic characteristics of this area reflect the historical life under the feudal Moslem rulers during the last centuries. Industrialization after the last war started to modernize the social conditions very quickly, especially in the Macedonian ethnic group, comprising during the last years more and more also the Moslem parts of the population. Different ethnic groups in Tetovo district live in the same areas, often in the same villages ; the villages vary in composition, from pure Macedonian, Turkish or Shqyptar to mixed in different proportions. Macedonians belong to the Eastern Orthodox Christian Church ; Shqyptars and Turks are Moslems. In spite of their geographical closeness there are major differences among them in regard to their social life, their habits and customs, and —most important for our present study—in regard to their hygienic habits in sexual life. The differences reflect the different historical and economic developments and the different religions.

The birth rates are high in all ethnic groups, being highest in Shqyptars, somewhat lower in Turks and lowest in Macedonians. Infant mortality is high in Macedonia (134 per thousand in 1951–1960) and in some areas of our study it was as high as 198 per thousand in 1961, the highest in Yugoslavia. Comparison with

* A national minority of Albanian origin.

Slovenia is illustrative in this regard, where the infant mortality for 1951–1960 was 53 per thousand.

There is a high incidence of tuberculosis, rickets and infectious disease in all three ethnic groups, only malaria was successfully controlled in the area.

The gynaecological pathology of the population is serious because of the underdeveloped medical service. There is no possibility for obstetric help in the majority of deliveries and one sees many serious complications such as ruptura uteri. There are marked differences in this regard among the ethnic groups, the situation being worst for Shqyptar, little better for Turkish and best for Macedonian women.

Dietary patterns differ substantially among them in regard to pork, pork fat and alcohol ; there are no restrictions for Macedonians, whilst Turks consume these items rarely and Shqyptars exceptionally or never.

Our Moslem group of women represents a heterogenous entity caused by differences in social behaviour and in strictness in the performance of religious practice in the sexual field. We show, therefore, separately the results of the gynaecological checkup on women coming for examination from a group of pure Shqyptar villages lying between the towns of Gostivar and Tetovo. We found the old Moslem type of life still preserved there and religion still represents the most important factor in both social and private spheres. Although covering the face is prohibited by law, the women still avail themselves of this habit when outdoors. They are not allowed to leave their courtyards without their husband's permission. Promiscuity is severely punished by the husband ; some would kill their wife for it. In the not so far past all brides were purchased by the bridegroom from the father and this custom is still widely practised ; therefore the husband treats his wife as his personal property. It is necessary to state that the males from these villages would also only very rarely permit promiscuity for themselves. Males and females shave their genital regions and they wash the genitalia obligatorily after intercourse. When there is no opportunity for washing they will abstain from intercourse. The men are circumcised. They marry early, the majority of brides being between their 16th and 19th birthdays. Divorces are very rare and so are multiple marriages. Concubinage is unknown. Polygamy, although allowed by religion, is practically nonexistent, probably for economic reasons. Birth rates are high and contraception, encouraged by the authorities, is very badly accepted, especially by males. Deliveries are conducted by older women at home in extremely unhygienic surroundings and there are many severe obstetric complications. Abortions are less common than in other ethnic groups and a higher proportion are spontaneous. The vast majority of males are occupied in their fields and in animal husbandry. Females don't work in the fields and are, with rare exceptions, all housewives. The population has an inadequate diet, although the villages are relatively well to do. This group is referred to as the " Moslem II " group.

The second part of our Moslem group is a mixture, about half being women of Turkish origin, the others being Shqyptar subjects from mixed villages (some of the them certainly not unlike their sisters in the villages). A substantial part of this group are inhabitants of mountainous villages on the Albanian border where the soil is poor and where males are obliged to go away for seasonal work. It often happens that the husband is absent for nine months in a year. Early marriages are common especially in Turkish women ; multiple marriages are not unknown and concubinage appears to be not exceptional. Birth rates are very high, and abortions are numerous and often artificial. Males and females wash their genitalia after intercourse and shave the genital regions. Males are circumcised. Promiscuity in males and also in females is much more common than one would have expected in an underdeveloped Moslem group. Females work in the fields and many are employed in local enterprises, particularly in making carpets of Persian type. This is the " Moslem I " group in our papers.

Our non-Moslem group is composed largely of Macedonians with a few Serbian, Croatian, Slovenian and other South Slavic representatives. This group has the highest percentage of employed women, others work only in the fields. Early marriages are common, the majority of brides are aged between 16 and 19 years. Births rates are high and so are abortions, which are often provoked. Modern obstetric care has started to penetrate among them. Males are engaged in agriculture and animal husbandry, but many of them work in local industry and in building. Seasonal workers are also not uncommon in this group. They do not practise circumcision at all and do not follow any hygienic prescriptions in sexual life.

### RESULTS OF THE GYNAECOLOGICAL EXAMINATIONS AND DISCUSSION

Gynaecological examination was performed by means of bimanual palpation, completed by necessary information in this field. Bacteriological tests in the cases of cervicitis and gonorrhoea were not made.

The results of the colposcopical and cytological examinations are shown in the preceding paper.

In the " anomalous development of the uterus " group all cases of infantile and hypoplastic uterus are included. All cases of retroflexion, descensus and prolapsus are included in the " abnormal position of the uterus " group. Cases of adnexitis and parametritis are shown together without special differentiation in regard to the aetiology of these findings. In the adnexal tumour group, cases of cystic tumours and inflammatory tumour formation, specific and nonspecific (tuberculous) are included. Detailed aetiological differentiation was impossible. In the group of anomalous menstruation no aetiological causes were identified and the same was true for sterility. We were not able to differentiate primary from the secondary sterilities.

The very low rates of cervicitis in the Moslem II group are intriguing. The results are in accordance with the observations of the authors during their work in the local gynaecological service, that vagina and cervix are extremely clean in Shqyptar women from the most religiously orthodox Moslem villages. One can hardly escape the impression that circumcision and/or hygienic practice connected with intercourse, have some influence in preventing vaginitis, cervicitis and other subsequent disorders.

Infantile and hypoplastic uterus might be connected with the general standard of living, including the inadequate diet, which is worst in the Moslem II group and best in the non-Moslem group. The high percentage of abnormal position of the uterus, descensus and prolapsus, is the consequence of the very high birth rates, deliveries being without skilled obstetric care in the majority of cases.

It is evident that the rates of parametritis and inflammatory tumours in the Moslem II group are low, a feature which might be connected with sexual hygiene or low abortion rates. The percentage of menstrual anomalies is also lowest in the Moslem II group.

TABLE I.—*Frequency of Benign Gynaecological Disorders*

| Group | Total No. of women | Cervicitis | | Tumour uteri | | Anomalous development of the uterus | | Abnormal position of the uterus | | Adnexitis and/or parametritis | | Tumour adnex. | | Anomalous menstruation | | Sterility | |
|---|---|---|---|---|---|---|---|---|---|---|---|---|---|---|---|---|---|
| | | No. | % | No. | % | No. | % | No. | % | No. | % | No. | % | No. | % | No. | % |
| Non-Moslem | 2555 | 73 | 2·8 | 34 | 1·3 | 105 | 4·1 | 758 | 29·7 | 361 | 14·1 | 66 | 2·6 | 204 | 8·0 | 217 | 8·5 |
| Moslem I | 540 | 19 | 3·5 | 15 | 2·8 | 32 | 5·9 | 182 | 33·7 | 101 | 18·7 | 30 | 5·5 | 75 | 13·9 | 136 | 25·2 |
| Moslem II | 538 | 9 | 1·7 | 10 | 1·8 | 45 | 8·4 | 136 | 25·3 | 28 | 5·2 | 4 | 0·7 | 25 | 4·6 | 58 | 10·8 |

SUMMARY

National, religious and socio-economic characteristics of three population groups in the Tetovo district of Macedonia, Yugoslavia, are described ; non-Moslems who do not follow any religious practice in sexual life, Moslems of mixed Turkish—Shqyptar composition with circumcised males and less rigorous practice of religious prescriptions, and inhabitants of a group of villages with a homogeneous Shqyptar population who follow religious prescriptions in sexual life very strictly.

From the analysis of benign gynaecological pathology in 3,633 women it is evident that the incidence of cervicitis, adnexitis, parametritis and similar conditions is lowest in the group of women who strictly follow Moslem religious prescription in sexual life and whose husbands are circumcised.